# Investigation on the Chronic Tension-Type Headache from the Persian Medicine Point of View

**DOI:** 10.31661/gmj.v8i0.1591

**Published:** 2019-10-31

**Authors:** Hossein Azizi, Asie Shojaii, Roshanak Ghods

**Affiliations:** ^1^Student Research Committee, Iran University of Medical Sciences, Tehran, Iran; ^2^Research Institute for Islamic and Complementary Medicine, School of Persian Medicine, Iran University of Medical Sciences, Tehran, Iran; ^3^School of Persian Medicine, Iran University of Medical Sciences, Tehran, Iran

**Keywords:** Chronic Tension-Type Headache, *Bayze*, *Soda’a*, Persian Medicine

## Dear Editor,

Tension-type headache (TTH) is the most prevalent primary headache disorder worldwide [1, 2]. According to the International Classification of Headache Disorders (ICHD-III) by the International Headache Society, TTH is classified into four categories: infrequent episodic, frequent episodic, chronic, and probable [3]. The chronic form, i.e., chronic TTH, is one of the types of headache mostly neglected to be treated, influencing directly on the patient’s quality of life, leading to a high economic burden on the society [1].

The typical symptoms of TTH generally include bilateral pressing or tightening with mild to moderate intensity (in terms of quality), with no deterioration in the normal physical activity, and is not associated with nausea or vomiting, but a possibility exists for the photophobia and phonophobia [3]. Chronic TTH, in addition to the above-mentioned symptoms, might persist from hours to days and also might be accompanied by mild nausea, as well as being repetitive. This pain might reduce working efficiency, leading to the absence from work and reducing the routine daily activities of the patient [4]. Due to the high prevalence and the possible effect on life and work quality, this type of headache has always been considered as a problem needing to be addressed. Persian medicine (PM) with a long history on treating different diseases according to four humors and temperaments may be a good choice for evaluating and finding a new treatment approach for this severe and chronic condition. In the reliable resources of PM, headache is known as “Soda’a,” and has been categorized into 28 types, including bilateral headache called “*Bayze”*[5]. This type of headache lasts for hours, and it is persisting. It is chronic, and its treatment is hard. It is sometimes called as *“Khoode”* because it engages all the head circumference like a helmet. This type of headache is caused by a minimum activity and is initiated by causes such as heat sources like sunlight and noise exposure, eating foods that produce gas, for example, onion and garlic. So, the patient hates the light and prefers to be alone in a quiet, comfortable, and dark environment. When the pain intensifies, the patient can not open the eyes, sometimes pain and tension are felt behind the eyes, and it would be annoying to touch the patients̓ head, and the pain is not pulsatory [5, 6]. According to the evaluation of headache in PM texts, seemingly “*Bayze”* may be the closest term for describing chronic TTH in PM. Also, there are several similarities guiding us to approach the chronic TTH, referred to as *Bayze* in PM (Table-1). According to the PM, vapors of thick humors or congestion of inappropriate humors between meningeal layers are reasons causing the *Bayze* headache accompanied by the pain while touching the head [6]. Based on the conventional medicine, this type of headache has an unknown cause, and its routine treatments are mostly symptomatic and palliative, including the analgesics, NSAIDs, antidepressants, anticonvulsants, muscle relaxants, and narcotics[7]. Considering the side effects of long-term administration of the drugs mentioned above, which could also lead to the overuse headache[4], PM recommendations for treatment of “*Bayze”* could be a good alternative or concurrent treatment for chronic TTH. The most important treatments suggested in PM texts include avoiding the consumption of the flatulent and thick food, using the laxatives, clearing the inappropriate humors from the brain through decoction of the *Cuscutaepithymum*, or *Lavandula stoechas*. After that, strengthening the brain and dislimning the vapors by smelling the *Nigella sativa* and *Origanum majorana*, and incensing the *Foeniculum vulgare*, avoid getting vapors into the brain by eating dry coriander blended with the grounded sugar, apple, quince, pear, hawthorn, *Rhus coriaria*, bathing at the fasting time, pouring the bitter almond oil in the ear, and continuous consumption of *Gol-e-ghand* (a blend containing *Rosa damascene* with sugar) along with *Terminalia chebula* in order to make a balance between the brain and stomach as two main underlying organs involving in this type of headache [6]. According to PM, the use of another traditional mixture mainly composed of *Emblica officinalis*, *T. bellerica,* and *T. chebula* called* as the “Etrifel-e-kabir”* is also recommended [5, 8]. Altogether, PM recommendations as an alternative or complementary treatment can give new ideas for treating this type of headache; of course, further research is needed for a precise evaluation in this regard.

## Conflict of Interest

None.

**Table 1:** Comparison of Symptoms of Chronic Tension-Type Headache and “Bayze” in Persian Medicine

**Chronic Tension-Type Headache**	**“** ***Bayze*** **” headache**	**References**
Without pulsation (pressing or tightening)	Non pulsatory	1, 3, 6, 7
Accompanied by photophobia and phonophobia	The affected person avoids the light and sounds	1, 3, 6, 7
Tenderness around skull aggravating by touching	Touching the head makes the patient uncomfortable	1, 3, 6, 7
Bilateral involvement	The pain surrounding the head	1, 3, 6, 7
Daily or very recurrent headache episodes	The headache begins with the smallest cause	1, 3, 6, 7
No definitive cure; treatments are mainly symptomatic and palliative	Hard to cure, hard to treat	1, 3, 6, 7
